# Proximate Analysis and Safety Profile of Farmed Edible Bird's Nest in Malaysia and Its Effect on Cancer Cells

**DOI:** 10.1155/2020/8068797

**Published:** 2020-01-21

**Authors:** Sin Nee Tan, Dahiru Sani, Chee Woei Lim, Aini Ideris, Johnson Stanslas, Christopher Thiam Seong Lim

**Affiliations:** ^1^Department of Medicine, Faculty of Medicine and Health Sciences, Universiti Putra Malaysia, Serdang, Malaysia; ^2^Faculty of Veterinary Medicine, Universiti Putra Malaysia, Serdang, Malaysia

## Abstract

Edible bird's nest (EBN) which is solidified swiftlet's saliva contains high nutritional value. It is widely consumed in countries like Malaysia, Indonesia, and Thailand. However, previous proximate analysis of Malaysia EBN was not representative of all the regions in Malaysia. In recent years, safety issues such as high nitrate and nitrite contents, presence of heavy metal, adulteration, fungal infection, and cancer cell stimulation were associated with EBN. Hence, this study aimed to determine the proximate analysis, safety profile during normal weather and hazy periods, and its effect on cancer cells stimulation in Malaysia-farmed EBN. Seven raw cleaned EBN samples were sourced from 6 different regions in Malaysia. Proximate analysis and safety profile were performed using official AOCA methods and Malaysian Standard. High protein (53.03–56.37%) and carbohydrate content (27.97–31.68%) with an acceptable level of moisture (10.8–14.04%) and ash (2.22–3.38%) were reported. A good safety profile was obtained with low nitrite and nitrate levels, with undetectable heavy metals and no significant growth of pathogenic microorganism except mould. Epidermal growth factor was detected but below the quantification level with the chicken EGF ELISA kit. The microculture tetrazolium (MTT) assay was performed for growth stimulation assessment comparing human EGF and EBN. There was no significant cell growth observed in cancer cells after EBN treatment. In conclusion, EBN Malaysia has a good nutritional profile, free of heavy metals, and an acceptable level of nitrate, nitrite, and microorganism profile except for mould contents. Furthermore, the *in vitro* study indicated that EBN was not associated with cancer cell growth.

## 1. Introduction

Edible Bird's Nest (EBN) is solidified saliva of *Aerodramus fuciphagus*. EBN has been used as the main ingredient in Traditional Chinese Medicine and cuisine as early as the Tang (618–907 AD) and Sung (960–1279 AD) dynasties [1]. EBN has been regarded as a luxurious local delicacy which contains high nutritional values and various medicinal properties [2]. High nutritional and medicinal values of EBN and the danger and difficulty of the nest collection process have made EBN as one of the world's most valuable animal food products consumed by mankind with an average selling price of $ 2500 per kilogram.

A few studies carried out looking into the proximate analyses of EBN in Malaysia [3, 4] and from neighbouring countries such as Indonesia [5] and Thailand [6] had concluded that EBN is a good source of protein, carbohydrate, and some trace elements. However, previous studies had reported a wide range of protein and carbohydrate contents from Malaysia EBN despite using the same method. Moreover, the analyses previously performed were not representative of all regions in Malaysia [3, 4, 7]. Despite being acclaimed as a nutritious food, there are many safety issues arising from Malaysia EBN in recent years; namely, high nitrate and nitrite contents [8, 9], EBN adulteration [5], presence of heavy metal [4, 10], pathological microorganism, and fungal infection [11]. The contaminants in EBN may be potentially harmful to the consumers. High intake of nitrate and nitrite were reported to be associated with an increased risk of stomach cancer [12]. The incident of Malaysia EBN was banned from exportation raised the public's concern about the high level of nitrite in EBN [8]. Meanwhile, microorganisms and fungal infections could lead to systemic or gastrointestinal infections [13]. Pigskin, karaya gum, Tremella fungus, and red seaweed are the common materials used in EBN adulteration for profit-making scams. It has proven to reduce EBN's nutritional value [5]. Moreover, Malaysia experiences severe haze pollution every year due to open burning. From the author's observation, the EBN collected during high Air Pollution Index (API) appeared to be dirtier and darker in colour. Hence, this study also wants to determine the nutritional and safety profile of the haze-affected EBN. This will be the first pilot study looking into the haze-affected EBN. In a nutshell, this paper aims to establish the proximate analysis and safety profile in terms of heavy metal, microorganism, and also nitrate and nitrite contents of EBN from different regions in Malaysia.

Some of the impressive medicinal properties of EBN identified include potent anti-inflammatory activity by suppressing the production of tumour necrosis factor alpha (TNF-*α*) [14], inhibition of influenza virus infection [15], cytoprotective properties from cyclophosphamide [16], neuroprotective effect [17], EGF like peptide which promotes cell proliferation [18], and estradiol hormones [19] which may be beneficial in women health by improving menstrual dysfunctions. EGF was first found to be present in EBN back in 1987. EGF in EBN was detected by using radioreceptor assay [18] and Western blot [20]. Human EGF is known to have the ability to stimulate cell growth and proliferation. It acts as a mitogenic factor that plays a growth-stimulating role in various epidermal and epithelial tissues. Hence, EBN is also deemed to have rejuvenating and antiaging properties. However, the receptor for EGF (EGFR) has been found highly expressed in various solid tumors. The dysregulation of EGF is also associated with the growth and progression of many cancers [21]. In addition, a previous paper had shown that EBN may stimulate colon cancer cell (Caco-2) growth *in vitro* [14]. Hence, this paper intends to quantify the amount of EGF in EBN and also looking into the possibility of proliferative effect of human cancer cells due to EBN consumption.

## 2. Materials and Methodology

### 2.1. Sample Collection and Preparation

**Figure 1:**
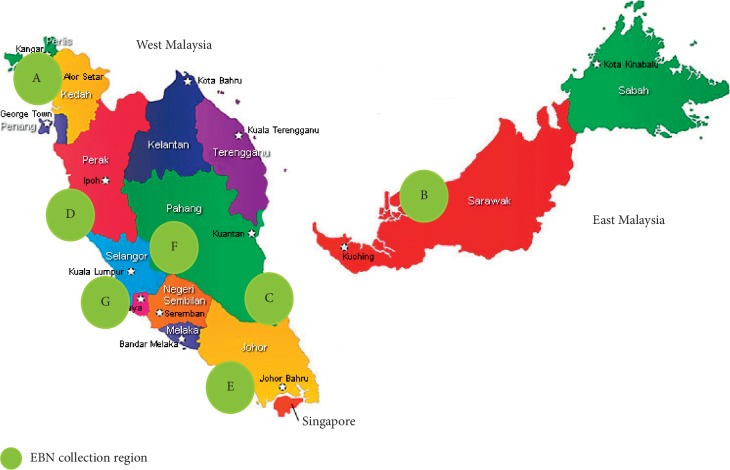
Seven raw, cleaned EBNs collected from different regions in Malaysia.

**Figure 2:**
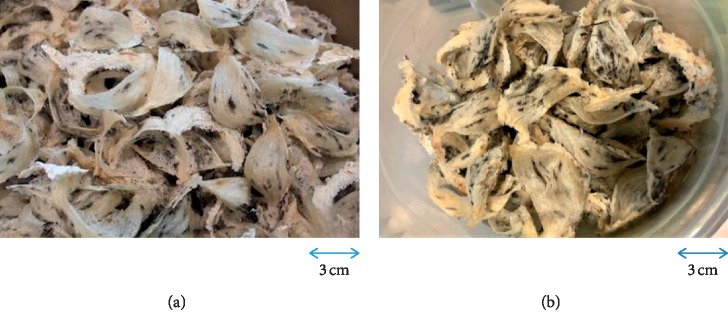
The physical appearance of raw, cleaned EBNs collected from Jerantut (F) with API <100 (a) and Port Klang (G) during the haze period with unhealthy API 100–200 (b).

Seven raw cleaned EBN samples were sourced from 6 different regions in Malaysia (Figure 1): A from Alor Setar, Kedah (northern region), B from Sibu, Sarawak (east region), C from Rompin, Pahang (east coast region), D from Kuala Selangor (west coast region), E from Johor Bahru (southern region), F from Jerantut, Pahang (central region), and G from Port Klang (west coast region). A total of 200 g bird nests were collected from a single bird farm in each region. The EBN was also collected specifically during the heavy haze period in March 2014 with an API of 100–200 for a month from Port Klang and compared with EBN collected from healthy API <100 level area in Jerantut. The EBN sample collected from Port Klang grossly appeared to be in darker colour likely due to heavy haze comparing the EBN sample collected from Jerantut during the same period of time (Figure 2). The EBN cleaning process was performed by the respective factories from different regions in Malaysia. Raw EBN was first soaked in clean water for 1 hour until the soft and glutinous material became partially loosened. The remaining feathers and dirt were removed manually using tweezers with the aid of magnifying glasses. The cleaned EBN was subsequently fan-dried at room temperature and shaped using a mould. All 7 samples of EBN were subsequently given a specific code and were blinded throughout the experiments: EBN 01 was C, EBN 02 was B, EBN 03 was E, EBN 04 was G, EBN 05 was A, EBN 06 was D, and EBN 07 was F.

### 2.2. Cell Lines

MCF-7 are human breast adenocarcinoma cells, A549 are human alveolar adenocarcinoma cells, Caco-2 are human epithelial colorectal adenocarcinoma cells, and HCT116 are human colorectal carcinoma cells which were purchased from American Tissue Culture Collection (ATCC) (Virginia, USA).

### 2.3. Chemical/Reagents

The pepsin and pancreatin enzymes were purchased from Nacalai Tesque Inc. (Kyoto, Japan). The chicken EGF ELISA kit was purchased from Cusabio Technology LLC (Houston, USA). Sodium hydrogen carbonate, sodium bicarbonate, hydrochloric acid, sodium hydroxide, foetal bovine serum (FBS), and biological-grade dimethylsulfoxide (DMSO) were purchased from Sigma-Aldrich (St. Louis, USA). Roswell Park Memorial Institute (RPMI) 1640 medium with L-glutamine, 2.5% trypsin (10x), and 10,000 U/mL penicillin–10 mg/mL streptomycin were purchased from GIBCO (New York, USA). Phosphate-buffered saline (PBS) was from Life Technologies (Maryland, USA) and 3-(4,5-dimethylthiazol-2-yl)-2,5-diphenyltetrazolium bromide (MTT) was from Molecular Probes (Oregon, USA). Human EGF (hEGF) was purchased from Pepro Tech Inc (New Jersey, USA).

### 2.4. Tissue Culture Materials

The tissue culture materials consisted of 25 cm^2^ and 75 cm^2^ plastic tissue culture flasks, 96-well flat-bottom tissue culture plates, 10 mL serological pipettes, and 15 mL plastic centrifuge tubes.

### 2.5. Proximate and Safety Profile Analysis

The analysis of total dry matter content of EBN was carried out by a food lab CHEMSIL (AIR & WATER) which was accredited under the Malaysia Accredited Laboratory Scheme ISO/IEC 17025. The official methods of the Association of Official Analytical Chemistry (AOAC) were employed to determine protein, carbohydrate, moisture, ash, and fat contents of the raw, cleaned EBN [22]. The crude protein content was determined by the Kjeldahl's method, using 6.25 as a conversion factor (AOAC Method 2001.11). Carbohydrate, free fat, moisture, and ash content were determined by AOAC 986.25E, Malaysia standard: 954 PART 5:2000, AOAC 950.46, and AOAC 923.03, respectively. The moisture content was determined by drying and heating the EBN at 105°C until a constant weight was obtained. The ash content was obtained by drying the EBN in a furnace at 550°C for 18 hours. The carbohydrate content was determined by the difference method (100% dry matter subtracting the percent of crude protein, ash, moisture, and fat). The method to determine nitrate and nitrite contents of EBN was based on the Malaysia Standard MS 2509:2012. A 20 *μ*L of EBN extract was injected into an ion chromatograph for analysis. Heavy metal content in EBN was analyzed using the In-House Method based on AOAC 999.11. EBN was dried and ashed at 450°C. 6 M HCL was added, and the solution was evaporated to dryness. The residue was dissolved in 0.1 M HNO_3_, and flame and graphite procedures were carried out to determine the heavy metal level. The microbiological compound was determined by the Australian Standard for *Escherichia coli*, *Samonella* spp., Coliform, and total plate count [23–25]. On the other hand, the official AOAC method was performed for *Staphylococcus aureus* content and Bacteriological Analytical Manual of Food and Drug Authority for mould and yeast content [26].

### 2.6. EBN Preparation

EBN extract was prepared as described by Goh et al. with modifications [27]. Briefly, about 1 g EBN was crushed to powder using pestle and mortar. The powdered EBN sample measured about 0.5–1 mm in size and was subsequently mixed with 100 mL of ultrapure water in a conical flask and mixed thoroughly. The mixture in the conical flask was then simmered in a pot (doubled boiled method) at 100°C in a water bath for 1 hour following the Chinese technique of cooking to preserve the taste. The extract was frozen in −80°C and subsequently freeze-dried into dry powder in a freeze dryer. It was stored at −20°C until required for further use.

### 2.7. Extract Digestion

The EBN extract subsequently underwent the pepsin-pancreatin enzyme digestive process [28]. Firstly, the pH of the EBN extract was adjusted to 2.0 with 1.0 M HCL. Pepsin was then added, and the mixture was incubated at 37°C for 2 hours in a shaking water bath. After that, the pH value was adjusted to 5.3 with 0.9 M·NaHCO_3_ solution and later to 7.5 with 1.0 M·NaOH. Subsequently, pancreatin was added to the mixture and was reincubated at 37°C for 2 hours. The reaction was terminated by keeping in the mixture in a test tube boiling for 10 minutes. The mixtures were cooled at room temperature, filtered, freeze-dried, and finally stored at −20°C until later use. The whole EBN extract preparation was performed mimicking the human digestive tract [19].

### 2.8. Preparation of Test Compound

Freeze-dry EBN was partly dissolved in ultrapure water and filtered with a syringe filter to make a stock solution with a concentration of 100 *μ*g/mL. It was stored at 4°C until required for further use.

### 2.9. Sample Concentration

Four mL of digested EBN extract was centrifuged with Amicon ultracentrifugal filters at 3000 rpm for 120 minutes with 3 minutes rest in between. The final volume for all EBN samples was 0.4 mL after concentration. The EBN sample was 10 times concentrated postprocedure.

### 2.10. ELISA Method

EGF assays were measured by using the 96-well plates (Cusaibo). 100 *μ*L of standard and EBN was added per well and incubated for 2 hours at 37°C. Ultrapure water served as the negative control. The liquid was removed. Then, 100 *μ*L of Biotin-antibody was added to each well and incubated for 1 hour at 37°C. Each well was aspirated and washed with 200 *μ*L of buffer for a total of three times. Followed by the addition of 100 *μ*L of horseradish peroxidase-avidin into each well, the plate was incubated for 1 hour at 37°C. The process of aspiration and washing was done for a total of 5 times. Then, 90 *μ*L of tetramethylbenzidine substrate was added to each well and incubated for 15–30 minutes at 37°C. 50 *μ*L of stop solution was subsequently added and thoroughly mixed. The absorbance at 450 nm and 570 nm were read using a Versamax microplate reader (Molecular Devices LLC, California, USA).

### 2.11. General Cell Culture Procedures

The cancer cells were cultured in a flask containing 5 mL of fresh medium at the subcultivation ratio of 1 : 4. The cultures were subsequently incubated at 37°C (5% CO_2_ and 95% air) until at least 80% of the flask was fully occupied, and then the subculture process was repeated. The cells were washed with phosphate-buffered saline (PBS), and 1 mL of trypsin-Ethylenediaminetetraacetic acid (EDTA) was added. After 5–10 minutes, the monolayer cells detached. 3 mL of complete growth medium was added to inactivate trypsin. Repeated gentle pipetting was applied to split the clumped cell apart. 0.5 − 1 × 10^6^ were subcultured into a new flask containing 10 mL of fresh medium and incubated at 37°C (5% CO_2_ and 95% air).

### 2.12. Plating

Once the cancer cells had subconfluency, they were detached by using trypsin-EDTA. The detached cells were then suspended in 6 mL of serum-free culture medium with repeated gentle pipetting technique. The cell concentration was determined with a haemocytometer. Two thousand cells in 0.18 mL culture medium were dispensed into each well of 96-well flat-bottom tissue culture plates.

The cell concentration was adjusted using the culture medium according to the following formula as follows:

(1) $$\begin{array}{c} M_{1}V_{1}\;=\;M_{2}V_{2}, \end{array}$$

where *M*_1_ = initial cell concentration, *V*_1_ = initial cell suspension volume, *M*_2_ = final cell concentration, and *V*_2_ = final cell suspension volume.

Using the multichannel pipette, 0.18 mL of cell suspension was dispensed into each well. The plate was incubated overnight at 37°C (5% CO_2_ and 95% air) before treatment.

### 2.13. Microculture Tetrazolium (MTT) Assay

The Caco-2, MCF-7, HCT116, and A549 cancer cell lines were tested with physiological concentrations of hEGF at 0.0001, 0.001, 0.01, 0.1, 1, 10, 100 ug/mL comparing with same concentration ranges of EBN 07 at 0.0001, 0.001, 0.01, 0.1, 1, 10, 100 *μ*g/mL. Two thousand cancer cells in 0.18 mL in serum-free medium were plated into each well of a 96-well plate and starved for 24 hours in the CO_2_ incubator. The cells in the culture plate were then directly visualized by using a microscope to ensure the cell had all attached to the wall of the well. The cells were then treated with 20 *μ*L of different concentrations of EBN and EGF as mentioned above. The cancer cells treated with 20 *μ*L of FBS 10% served as the positive control, while the cancer cells treated with 20 *μ*L of distilled water served as the negative control. The cells were continued to incubate for 24, 48, and 72 hours.

### 2.14. MTT Cell Viability Assay

After treatment, 50 *μ*L of MTT stock solution (2 mg/mL) was added into each well. Then the culture was incubated for another 4 hours. The mixture of culture medium and MTT solution was aspirated, and the formazan crystals formed were solubilized with 100 *μ*L of analytical-grade DMSO. The absorbance of the formazan solution at a wavelength of 450 nm was measured by using the Versamax microplate reader. The cell viability at time-zero was evaluated at 24, 48, and 72 hours postseeding, of which absorbance was measured.

The percent of cell growth was calculated by using the following formula:

(2) $$\begin{array}{c} \frac{\left(\text{AT}\right)}{\left(\text{AC}\right)}\;\times \;100\text{,} \end{array}$$

where AT =  absorbance of treatment groups at 24, 48, and 72 hours posttreatment and AC = absorbance of the vehicle control group at 24, 48, and 72 hours after treatment.

The values of the cell viability were presented as the mean triplicate (*n* = 3) ± standard deviation (SD).

### 2.15. Statistical Analysis

The data are represented as mean ± standard deviation. Statistical Package for Social Sciences (SPSS) version 22 software by IBM (New York, USA) was performed to determine statistically significant differences. One-way and two-way analyses of variance (ANOVA) were used in the statistical analysis, in which Bonferroni post hoc test with a confidence interval adjustment of least significant difference was applied. Statistically significant differences were determined by *p* < 0.05.

## 3. Results and Discussions

### 3.1. Proximate Analysis

In this study, the order of EBN composition (from highest to lowest) is protein, carbohydrate, moisture, ash, and lipid (Table 1). The protein content ranges from 53%–56%, which were lesser than EBN collected in Thailand [6] and a local study [3] (60.9%–66.9% and 56%–61.5%, respectively). In contrast, our protein content range was higher than those collected from Penang and Indonesia which was 24%–49% [7] and in Batu Pahat, Johor which was 35.8% [4] by using the same method for protein analysis. A previous study reported the overall crude protein content could reduce by 1.1–6.2% with the incorporation of adulterant products [5]. Our result had shown good consistency with no significant differences in terms of protein content in EBN from different regions in Malaysia. This can be possibly explained by the fact that the saliva was produced by the same species of swiftlet even though they have different habitat environments. The total carbohydrate content in this study ranged from 27.97%–31.68%. This finding was close to a study on the nutritional composition of the farmed EBN in Thailand with the carbohydrate content ranging from 25.4%–31.4% [6]. The crude fat levels in all samples were consistent at 0.1%, which was the lowest in comparison with previous papers [5–7, 29]. The amount of fat content present in EBN was too small in amount, and its function is still unclear. The moisture content of all collected EBN samples was within the tolerance level (<15%), ranging from 10.80% to 14.04%. The moisture level was likely affected by the cleaning and drying process of EBN which varies from one factory to another factory. The EBN storage method may also affect the moisture level. Lastly, the ash content ranged from 2.22–3.38%, which was relatively low compared with other studies which were ranging from 2.1%-7.3% and 2.75%–7.5%, respectively [5, 7, 29]. The higher level of ash may be indicating the cleaning process may not be done thoroughly. Even though the EBNs were collected from 6 different regions in Malaysia, but it was only collected from a single bird farm due to financial constraints. Hence, the result may not be representative of the whole regions in this study.

### 3.2. Safety Profile: Nitrate and Nitrite

There were significant differences in the nitrite level of EBN collected from different regions in Malaysia (Table 1). The highest nitrate and nitrite amount were found in EBN from region C, but the readings were still within the tolerance level. The differences were likely attributed to the different bird farm environments such as humidity, pH and climate, contamination during harvesting, or the cleaning process of the collected EBN. There was no previous study done in comparison with nitrite and nitrate contents of the farmed EBN from different regions. Most studies were comparing farmed EBN with wild EBN. Paydar et al. reported cave EBN contained 5-fold higher nitrite content and 297-fold higher nitrate content than those in the farmed EBN [8], while another recent study found that the cave EBN contained 148-fold higher amount of nitrite and 367-fold higher amount of nitrate than the farmed EBN [9]. The finding was explained by the differences between the cave and swiftlet house environment, as the swiftlet soil and guano were constantly removed from the swiftlet house. Good ventilation in the birdhouse also helps to reduce bacterial anaerobic fermentation process. Traditionally, the red EBN also known as premium swiftlet's blood EBN had a very high market value compared with the white nest due to limited supply and was believed to have higher medicinal and nutritional value [8]. A few recent studies had shown that the redness of EBN can be attributed to nitrate and nitrite contamination in EBN [8, 9, 30]. Nitrite, a nitrate's metabolite, has been commonly used as food additive or preservative. However, it is considered hazardous if the amount exceeded the daily allowance. Nitrate and nitrite itself are not carcinogenic, but nitrite may react with some dietary amine compounds to form carcinogenic nitrosamines which can cause stomach cancer [12]. Farmed EBN in Malaysia in this study appeared whitish in colour (Figure 2). Hence, it was not surprising that both the nitrate and nitrite level were not exceeding the tolerance level.

### 3.3. Safety Profile: Heavy Metal

Malaysia EBN from the regions studied was free from heavy metal (Table 1). The findings were similar to a local study done by Lee et al. who reported there was no detectable cadmium and mercury. But, there was presence of trace amounts of arsenic (0.0237 mg/kg) and lead (0.0203 mg/kg) [4]. On the other hand, another local study reported that 1 out of the 10 tested EBN samples had a high level of mercury in a raw, uncleaned EBN sample from Kluang [10]. Heavy metal contamination in EBN could be from within the swiftlet house farms such as rusty iron bars, lead-based paints, and mercury-contaminated water supply or during the processing or manufacturing processes of EBN. The chemicals and materials used could also be a source of heavy metals [10]. Heavy metals cannot be biodegraded. They were known to have long-term cumulative effects in humans, causing various diseases and disorders even at relatively low concentrations [31]. Therefore, it is important to study the levels of these elements in EBN to ensure the safety profile.

### 3.4. Safety Profile: Microbiology Profile

There was very limited literature describing the microbiology profile of EBN. *Escherichia coli*, Coliform, *Staphylococcus aureus*, and *Salmonella* spp. which may cause serious gastrointestinal infections to humans [13] after consumption were absent in all EBN samples (Table 1). The safety profile in terms of microorganism was further supported by the low total plate count in EBN from all regions. Mould is a type of fungus that is multicellular and grows in hyphae, while yeast is a type of single-cell fungus. Both organisms belonged to the Fungi kingdom. All EBN samples had a high level of mould except the EBN from region A. Meanwhile, yeast content for all EBN samples was within the tolerance level. A study done found that the amount of yeast and mould had exceeded the limit set by SIRIM for all EBN [11]. The common fungi that isolated were *Aspergillus* sp., *Candida* sp., *Cladosporium* sp., *Neurospora* sp., and *Penicillium* sp. In the same study, there was no significant difference (*p* > 0.05) in the reduction of the number of fungi even after boiling the EBN. The high level of fungus growth may be due to the environment of the bird farm which has a lower temperature, with high-level humidity or moisture. The fungus is usually benign and rarely causing serious infection in an immunocompetent host. However, EBN has been recognized as prestigious food with multiple medicinal properties. Hence, many consumers who have medical illness or in the immunocompromised state, for example, malignancy, on long-term steroid, diabetes mellitus, or organ transplant recipients may develop severe opportunistic fungal infections after consuming EBN. Further studies are needed to determine the effect of fungus growth in EBN on humans. Besides that, an effective method to remove fungal contaminations in EBN needs to be developed for safe consumption. With the finding of high level of mould, this issue should be highlighted, and prompt action is necessary. The presence of these microorganisms may jeopardize the quality of EBN and pose health risks to consumers. Hence, the standards of EBN should be monitored regularly to ensure the quality of EBN and consumer safety.

### 3.5. Comparing EBN Collected during the Haze Period

In this study, there are differences in terms of physical appearance as the EBN collected from Port Klang during API 100–200 appeared to be darker and dirtier (Figure 2). There was no significant difference in protein, carbohydrate, ash, crude fat, moisture, and nitrate and nitrite contents (Table 1). EBN from both areas has similar heavy metal and microbiology profiles (Table 1). The same result was also observed when comparing with other EBN collected from other different regions without air pollution period which we had reported earlier. Therefore, there were no significant differences found in terms of nutritional profile, heavy metal, and microbiology profile of these haze-contaminated samples.

### 3.6. EGF Quantification

There was the presence of 30.7 pg/mL and 74.5 pg/mL of EFG in crude EBN 01 and EBN 02, respectively (Table 2). However, both of the detected levels were below the quantification limit of the ELISA kits (125 pg/mL–8000 pg/mL). There was no detected EGF found in all digested EBNs and digested EBNs from three selected regions (EBN 01, 02, and 07), despite the post 10 times concentration of the EBN (Table 2). The 3 selected samples went ahead with the ultracentrifugal filter process given the low level of EGF detected in crude EBN 01 and EBN 02 while EBN 07 was selected for cancer cell work. The result had shown the presence of EGF in EBN. However, we failed to quantify the amount of EGF in all EBN samples by using the ELISA quantification assay. A few factors were identified which affected the result. First of all, the protein may be denatured during the EBN cooking process. Subsequently, during the enzyme digestion process, EBN was undergoing artificial digestion pathway to mimic the human digestive tract which may further alter the protein nature. This may be explained by the ELISA result whereby there was a detection of EGF in crude EBN but not found in digested EBN. Secondly, freeze-dried EBN appeared to be fine pale powder, but it became thick gelatinous liquid again after adding a small amount of ultrapure water. Hence, it required a large amount of ultrapure water to partly dissolve the freeze-dried EBN leading to the possibility of overdiluted sample. Thirdly, freshly prepared EBN extract after the boiling process appeared to be like EBN soup which contained EBN aqueous form and insoluble jelly-like substance. Only the aqueous form of EBN was able to be taken up by the pipetting method. Thereby, we postulated that EGF may have bound tightly to the insoluble jelly-like substance leading to underestimation of EGF. Insufficient extraction was previously highlighted as one of the reasons for low detection of EGF content in EBN [18]. Furthermore, Yang et al. had identified that only one kind of EGF antibody can specifically bound to the EGF of EBN after trial of multiple types of EGF primary antibodies [20]. They had used an anti-EGF primary antibody and human anti-rabbit immunoglobulin conjugate as a secondary antibody in the immunoblotting assay. Similar to our present study, the ELISA kits used contained rabbit derived polyclonal antibody for chicken EGF detection. However, no previous study had used this chicken EGF ELISA kits in EGF detection for EBN. Therefore in future studies, we suggest the use of alternative quantification methods such as Western blot or radioreceptor assay as the previous study had proven the presence of EGF in EBN even though in a very small amount [18, 20].

### 3.7. The Effect of EBN on the Growth of Cancer Cells

**Figure 3:**
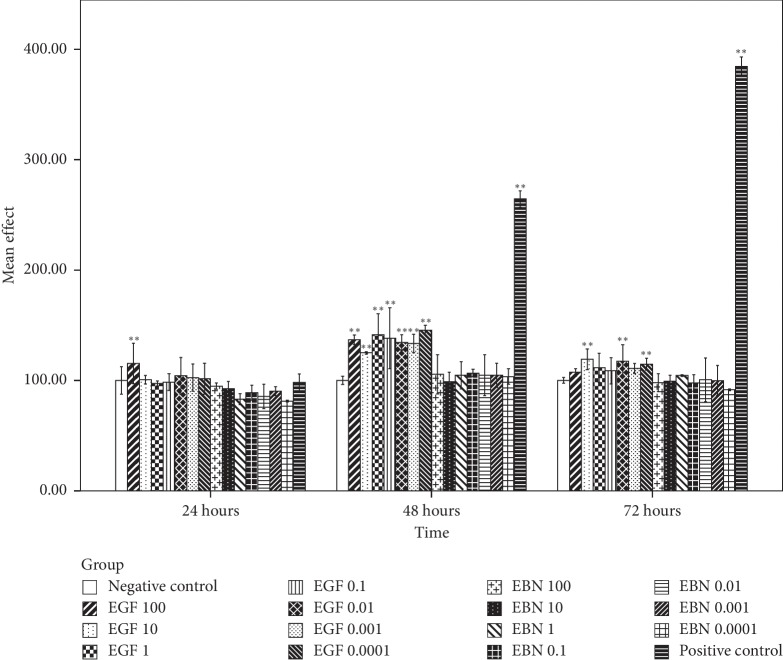
Caco-2 cell viability posttreatment with different concentrations of hEGF (0.0001, 0.001, 0.01, 0.1, 1, 10, and 100 *μ*g/mL) and EBN (0.0001, 0.001, 0.01, 0.1, 1, 10, and 100 *μ*g/mL) at 24, 48, and 72 hours by an assessment of cell viability using MTT assay. Cell viability is presented as mean (*n* = 3) ± SD. Statistically significant differences were determined by two-way ANOVA with Bonferroni post hoc test, and a confidence interval adjustment of least significant difference was applied; *p* < 0.05 (^*∗*^) and *p* < 0.01 (^*∗∗*^) compared with the negative control (ultrapure water). The positive control was treated with FBS.

**Figure 4:**
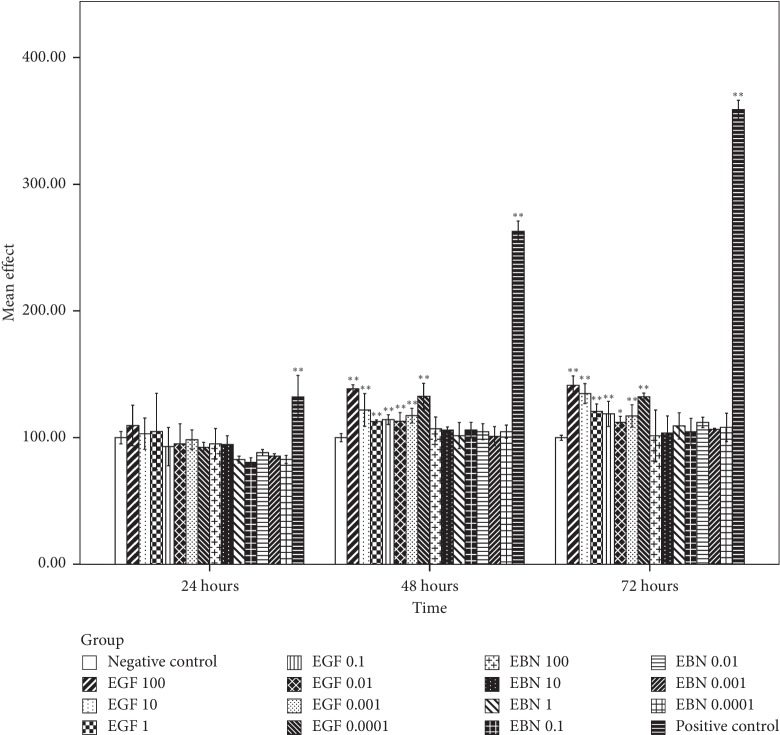
MCF-7 cell viability posttreatment with different concentrations of hEGF (0.0001, 0.001, 0.01, 0.1, 1, 10, and 100 *μ*g/mL) and EBN (0.0001, 0.001, 0.01, 0.1, 1, 10, and 100 *μ*g/mL) at 24, 48, and 72 hours by an assessment of cell viability using MTT assay. Cell viability is presented as mean (*n* = 3) ± SD. Statistically significant differences were determined by two-way ANOVA with Bonferroni post hoc test, and a confidence interval adjustment of least significant difference was applied; *p* < 0.05 (^*∗*^) and *p* < 0.01 (^*∗∗*^) compared with the negative control (ultrapure water). The positive control was treated with FBS.

**Figure 5:**
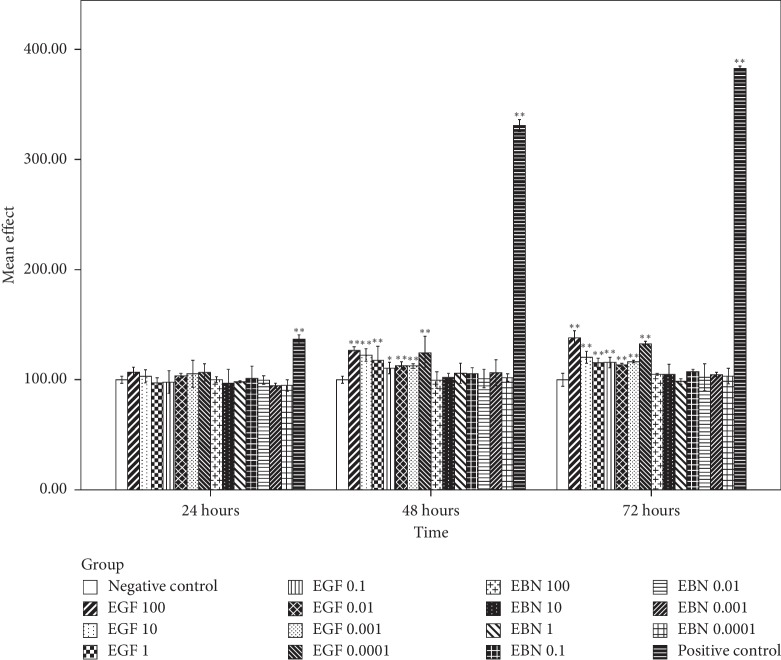
HCT 116 cell viability posttreatment with different concentrations of hEGF (0.0001, 0.001, 0.01, 0.1, 1, 10, and 100 *μ*g/mL) and EBN (0.0001, 0.001, 0.01, 0.1, 1, 10, and 100 *μ*g/mL) at 24, 48, and 72 hours by an assessment of cell viability using MTT assay. Cell viability is presented as mean (*n* = 3) ± SD. Statistically significant differences were determined by two-way ANOVA with Bonferroni post hoc test, and a confidence interval adjustment of least significant difference was applied; *p* < 0.05 (^*∗*^) and *p* < 0.01 (^*∗∗*^) compared with the negative control (ultrapure water). The positive control was treated with FBS.

**Figure 6:**
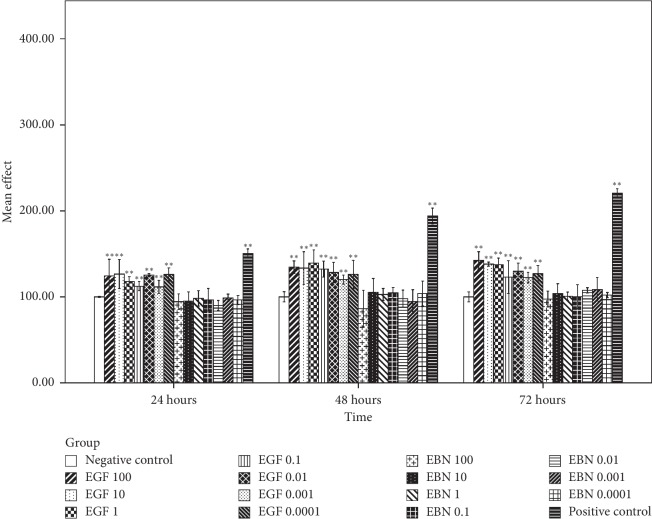
A549 cell viability posttreatment with different concentrations of hEGF (0.0001, 0.001, 0.01, 0.1, 1, 10, and 100 *μ*g/mL) and EBN (0.0001, 0.001, 0.01, 0.1, 1, 10, and 100 *μ*g/mL) at 24, 48, and 72 hours by an assessment of cell viability using MTT assay. Cell viability is presented as mean (*n* = 3) ± SD. Statistically significant differences were determined by two-way ANOVA with Bonferroni post hoc test, and a confidence interval adjustment of least significant difference was applied; *p* < 0.05 (^*∗*^) and *p* < 0.01 (^*∗∗*^) compared with the negative control (ultrapure water). The positive control was treated with FBS.

Based on Figure 3, the first significant post-hEGF treatment-induced Caco-2 cell growth was observed at the highest concentration of hEGF, 100 *μ*g/mL, during the first 24 hours incubation period. It was followed by significant cell growth in all concentrations of hEGF treatment at 48 hours and with a concentration of 10, 0.01, and 0.0001 *μ*g/mL at the incubation period of 72 hours. Cell growth with hEGF as high as 50% compared with negative control was observed. There was no significant Caco-2 cell growth after being treated with various concentrations of EBN at different intervals of time. MCF-7 and HCT116 cells that were treated with hEGF across all concentrations have significant cell growth at 48 and 72 hours. In contrast, no significant cell growth was seen after being treated with various concentrations of EBN (Figures 4 and 5). There was significant A549 cell growth after being treated with all ranges of hEGF concentrations at 24, 48, and 72 hours but no significant A549 cell growth after EBN treatment (Figure 6). A positive control cell was treated with FBS which showed marked significant cell growth. There was no significant cancer cell stimulation in all cancer cells after being treated with a wide range of concentrations from 100–0.0001 *μ*g/mL EBN extracts. However, most of the cancer cells grew well with the addition of hEGF indicating the presence of EGF does stimulate cancer cells growth. Since the discovery of EGF in EBN, there are very limited studies regarding the function and medicinal properties of the avian EGF. The human and avian EGFR was found to contain a complete coding sequence and was highly homologous, about 78% identical after the sequence of a cDNA clone in the primary structure of the chicken EGF receptor. However, the Basic Local Alignment Search Tool (BLAST) by the National Centre of Biotechnology Information was used to compare the human EGF amino acid sequence with the chicken EGF amino acid sequence and was found only at 36% identical. There was only 1 previous study showing significant dose-dependent growth of Caco-2 cells after being treated with EBN [14]. However, their postulation for the cancer cell growth factor was due to the presence of sialic acid in EBN instead of EGF. Besides that, the present of adulterants incorporated in EBN [5] may also be the reason for cancer cell stimulation. The percentage of cell proliferation comparing the control was as high as 215%. In the contrary, there was no significant cancer cell growth despite being treated with a high or low concentration of EBN in this study. Our study findings were supported by Roh et al. reported that there were no observable effects on MCF-7 and Hep2B, human liver cancer [32]. The same study found that EBN has properties of promoting 34% and 38% increase in the cell proliferation rate of normal hADSCs and NHFs, respectively. Hence, they concluded that EBN was shown to affect normal human cells, but not the transformed cell line. In our study, the 4 selected types of cancer cells are according to the presence of EGF mutation in these particular cancer cells. In modern medicine, tyrosine kinase inhibitor as a cancer treatment for lung, colon, and breast carcinoma is well established. Hence, we postulated that the selected cancer cells may likely to have growth simulation by the presence of EGF content in EBN. There are a few factors that may affect the results. The protein or EGF extraction method may not be adequate to release all the relevant bioactive substances. Besides that, the content of EGF which present in EBN may be too minute to stimulate cancer cell growth, and the amount cannot compare with the same concentration of pure hEGF. There was only 36% identical amino acid sequence between hEGF and avian EGF which could explain why the EBN is unable to stimulate human cancer cells like hEGF. More studies are needed to research upon the proper extraction methods of pure EGF from EBN and quantification of EGF contents in order to carry out more precise experiments.

## 4. Conclusion

This study demonstrated that raw, cleaned EBN from different regions in Malaysia has good consistency in their proximate analysis, even during hazy periods. They contained high proteins and carbohydrates with low fat and ash content. Besides that, EBN is also safe to be consumed as no heavy metals were detected, with acceptable microbiology profile and the nitrite level within standards set by the government except the presence of mould above the tolerance level. EGF was detected in EBN, but the amount was below the quantification level via the ELISA method. Our results had demonstrated no significant cancer cell stimulation after being treated with different concentrations of EBN extract. Hence, EBN is safe to be consumed by the human in terms of carcinogenic risk.

## Figures and Tables

**Table 1:** Proximate composition, safety profile, and microorganism profile of the different samples of EBN collected from different regions in Malaysia (A from Alor Setar, Kedah; B from Sibu, Sarawak; C from Rompin, Pahang; D from Kuala Selangor; E from Johor Bahru; F from Jerantut, Pahang; and G from Port Klang, Selangor).

Parameters	Regions							Tolerance level
	A	B	C	D	E	F^*∗*^	G^£^	
Proximate analysis								
Protein	54.3 ± 1.9^a^	53.9 ± 1.9^a^	53.0 ± 0.7^a^	53.7 ± 1.1^a^	54.4 ± 3.8^a^	55.5 ± 2.5^a^	56.4 ± 2.8^a^	NA
Carbohydrate	29.7 ± 4.8^b^	30.5 ± 5.5^b^	28.0 ± 7.0^b^	31.7 ± 3.5^b^	28.6 ± 4.6^b^	28.6 ± 6.0^b^	28.8 ± 6.2^b^	NA
Moisture	10.8 ± 0.8^c^	12.4 ± 1.4^c^	12.3 ± 1.2^c^	13.1 ± 0.3^c^	14.0 ± 0.5^c^	12.1 ± 1.6^c^	12.1 ± 1.2^c^	<15%
Ash	2.8 ± 0.2^d^	2.7 ± 0.2^d^	3.4 ± 0.7^a^	2.7 ± 0.1^d^	2.9 ± 0.3^d^	2.8 ± 0.1^d^	2.2 ± 0.1^a^	NA
Crude fat	0.1	0.1	0.1	0.1	0.1	0.1	0.1	NA

Safety profile								
Nitrate	24.9 ± 0.5^a^	39.4 ± 1.0^b^	52.6 ± 0.9^c^	47.0 ± 0.6^d^	41.5 ± 0.5^e^	31.1 ± 0.5^f^	35.9 ± 0.1^g^	NA
Nitrite	10.1 ± 0.4^a^	10.4 ± 0.2^a^	18.4 ± 0.4^b^	15.8 ± 0.1^c^	11.0 ± 0.2^d^	10.3 ± 0.1^a^	11.4 ± 0.2^e^	<30

Heavy metal								
Arsenic	<0.01	<0.01	<0.01	<0.01	<0.01	<0.01	<0.01	<2
Mercury	<0.01	<0.01	<0.01	<0.01	<0.01	<0.01	<0.01	<1
Lead	<0.02	<0.02	<0.02	<0.02	<0.02	<0.02	<0.02	<0.05
Cadmium	<0.01	<0.01	<0.01	<0.01	<0.01	<0.01	<0.01	<1
								<100

Microorganism								
*E. Coli*	ND	ND	ND	ND	ND	ND	ND	NA
*S. Aureus*	ND	ND	ND	ND	ND	ND	ND	<100
*Salmonella*	ND	ND	ND	ND	ND	ND	ND	D/ND
Coliform	43	20	ND	ND	ND	ND	23	<1100
Mould	40	20	140	40	<10	20	30	<10
Yeast	10	<10	<10	<10	<10	<10	<10	<10
Total plate count	12 × 10^5^	9.5 × 10^5^	5 × 10^5^	2.3 × 10^5^	3.2 × 10^5^	1.8 × 10^5^	16 × 10^5^	<25 × 10^5^

Mean ± SD (*n* = 3). ND = not detected; D = detected; NA = not applicable. ^*∗*^EBN collected during API 100–200 (polluted air); ^£^EBN collect during API < 100. Proximate analysis results were expressed in g/100 g of EBN; safety profile results were expressed in mg/kg of EBN; microorganism profile results were expressed in MPN/g or CFU/g of EBN on the dry basis. ^a–g^mean in category row with the same letter are not significantly different (*p* > 0.05).

**Table 2:** Chicken EGF ELISA quantification results of seven crude and digested EBN samples from six different regions in Malaysia and post-ultracentrifugal filter of the digested EBN from three selected regions (EBN 01, 02, and 07).

	Crude sample concentration (pg/mL)	Digested sample concentration (pg/mL)	Digested sample post-ultracentrifugal filter concentration (pg/mL)
Negative control	0	0	NA
EBN 01	30.7	0	0
EBN 02	74.5	0	0
EBN 03	0	0	NA
EBN 04	0	0	NA
EBN 05	0	0	NA
EBN 06	0	0	NA
EBN 07	0	0	0

Mean ± SD (*n* = 3). NA: not applicable. EBN 01 from Rompin, Pahang; EBN 02 from Sibu, Sarawak; EBN 03 from Johor Bahru, Johor; EBN 04 from Port Klang, Selangor; EBN 05 from Alor setar, Kedah; EBN 06 from Kuala Selangor, Selangor; EBN 07 from Jerantut, Pahang. Results expressed in pg/mL of EGF. The detection range of chicken EGF ELISA kit: 125–8000 pg/mL.

## Data Availability

The data used to support the findings of this study are available from the corresponding author upon request.
